# Effects of acute selective cyclooxygenase‐2 inhibition on skeletal muscle microvascular oxygenation and exercise tolerance

**DOI:** 10.1113/EP092518

**Published:** 2025-03-30

**Authors:** Michael D. Belbis, Michael J. Holmes, Joseph Yao, Christopher W. Kinnick, Christopher K. Kargl, Carly Day, Nicole L. Noel, Timothy P. Gavin, Bruno T. Roseguini, Daniel M. Hirai

**Affiliations:** ^1^ Department of Health and Kinesiology Purdue University West Lafayette Indiana USA; ^2^ Department of Exercise Science Aurora University Aurora Illinois USA; ^3^ Athletics Department Purdue University West Lafayette Indiana USA; ^4^ Department of Pharmacy Practice Purdue University West Lafayette Indiana USA

**Keywords:** COX‐2, microcirculation, NIRS, oxygen uptake kinetics, pulmonary ${\dot{V}}_{O_{2}}$

## Abstract

The cyclooxygenase (COX) pathway regulates vascular tone and, therefore, local O_2_ delivery‐utilization matching. The two main isoforms, COX‐1 and COX‐2, may promote opposing effects on contracting muscle O_2_ transport in health by inducing vasoconstriction and vasodilatation, respectively. Whether COX‐2 and its main vasodilatory product (prostacyclin, PGI_2_) modulate microvascular O_2_ transport to skeletal muscle and thus exercise tolerance is unknown. We tested the hypothesis that acute selective COX‐2 inhibition (SC2I) would impair cardiorespiratory and skeletal muscle microvascular responses from rest to exercise, thereby reducing exercise tolerance in healthy adults. Twelve individuals participated in a randomized, double‐blind, crossover study to receive SC2I (200 mg celecoxib) or placebo (control, CON). Moderate and severe intensity cycling were performed with measurements of heart rate, arterial blood pressure, pulmonary oxygen uptake (${\dot{V}}_{O_{2}}$), leg muscle microvascular oxygenation ($S_{tO_{2}}$; near‐infrared spectroscopy) and time to exhaustion. Leg muscle $S_{tO_{2}}$ was also assessed during cuff occlusion protocols. SC2I decreased the plasma concentration of the stable PGI_2_ metabolite 6‐keto prostaglandin F_1α_ (CON: 203 (54) pg/mL; SC2I: 108 (54) pg/mL; *P* = 0.002). There was no difference in exercise tolerance (CON: 278 (55) s; SC2I: 298 (75) s), arterial blood pressure, heart rate, pulmonary ${\dot{V}}_{O_{2}}$ or leg muscle $S_{tO_{2}}$ from rest to moderate or severe exercise between conditions (*P *> 0.05 for all). Moreover, there was no significant difference in $S_{tO_{2}}$ during cuff occlusion protocols between conditions. Contrary to our hypothesis, these data indicate that COX‐2 is not obligatory for the regulation of skeletal muscle microvascular oxygenation at rest or during moderate or severe intensity exercise, and therefore does not modulate exercise tolerance in healthy adults.

## Abstract (highlights)

- **What is the central question of this study?**
  What are the effects of acute selective cyclooxygenase‐2 (COX‐2) inhibition via oral celecoxib on cardiorespiratory and skeletal muscle microvascular function at rest and during moderate and severe intensity exercise in healthy young individuals?
- **What is the main finding and its importance?**
  Selective COX‐2 inhibition reduced plasma prostacyclin metabolites but had no effect on central or peripheral determinants of oxygen transport, peak oxygen uptake or exercise tolerance. These findings indicate that COX‐2 is not obligatory for skeletal muscle oxygen delivery–utilization matching at rest or during contractions in healthy adults.

## 1 INTRODUCTION

Skeletal muscle microvascular oxygenation is determined by the matching between oxygen (O_2_) delivery and utilization (Behnke et al., 2001; Hirai et al., 2018; McDonough et al., 2001). Due to limited and finite O_2_ stores in the human body, fine‐tuned changes in O_2_ transport within the cardiorespiratory system are required to support alterations in metabolic demand such as those associated with exercise and activities of daily living (Joyner & Casey, 2015). Among other mechanisms, the regulation of local O_2_ delivery during exercise involves a complex interplay of vasodilators that act synergistically (Joyner & Wilkins, 2007). The cyclooxygenase (COX) enzyme system participates in this redundant control via the production of vasodilating prostanoids (Boushel et al., 2004), but its relevance to increased O_2_ delivery during dynamic muscle contractions remains controversial (Aiku & Marshall, 2019). Prostacyclin (PGI_2_) is the primary vasodilator produced by the COX system in the vascular endothelium, especially under hypoxic or low $P_{O_{2}}$ conditions, such as those present in contracting skeletal muscle (Busse et al., 1984; Michiels et al., 1993). While PGI_2_ is released locally during dynamic muscle contractions in proportion to exercise intensity (Boushel et al., 2002; Karamouzis et al., 2001), previous non‐selective inhibition of the COX pathway has reduced O_2_ delivery during muscle contractions in some instances (Cowley et al., 1985; Duffy et al., 1999, 1998; Herbaczynska‐Cedro et al., 1976; Janczewska & Herbaczyńska‐Cedro, 1974; Kilbom & Wennmalm, 1976; Murrant et al., 2014; Schrage et al., 2004; Wilson & Kapoor, 1993; Win & Marshall, 2005), but not all (Beaty & Donald, 1979; Casey & Joyner, 2011; Crecelius et al., 2011; Einstein & Goodman, 1980; Lopez et al., 2013; Mortensen et al., 2007; Nowak & Wennmalm, 1979; Shoemaker et al., 1996; Young & Sparks, 1980).

The controversial role of vasodilating prostanoids in regulating oxygen transport to contracting muscle may stem, at least in part, from the reliance on non‐selective pharmacological COX inhibitors as research tools. The COX enzymes (also known as prostaglandin G/H synthases) comprise at least two main isoforms (COX‐1 and COX‐2) which participate in the conversion of arachidonic acid to prostaglandin G_2_ and prostaglandin H_2_. The latter prostaglandins can be subsequently metabolized by downstream synthases coupled preferentially with COX‐1 or COX‐2 to generate prostanoids such as the vasodilator PGI_2_ as mentioned above, and the vasoconstrictor thromboxane A_2_ (TxA_2_) (Félétou et al., 2011; Trappe & Liu, 2013; Wang et al., 2021). It is now well appreciated that both COX‐1 and COX‐2 are expressed constitutively and can be upregulated, for instance, by shear stress among other processes (Doroudi et al., 2000; Félétou et al., 2011; Funk & FitzGerald, 2007). While COX‐1 promotes mainly vasoconstriction and platelet aggregation via TxA_2_ synthesis, COX‐2 is responsible for most of the generation of PGI_2_ under physiological conditions thereby promoting vasodilatation in healthy humans (Funk & FitzGerald, 2007; McAdam et al., 1999; Wang et al., 2021). Within this context, non‐selective COX inhibition can interfere with the synthesis of both the vasoconstrictor TxA_2_ (by blocking COX‐1) and the vasodilator PGI_2_ (by blocking COX‐2). On the other hand, selective COX inhibition can shift the TxA_2_/PGI_2_ balance and thus potentially enhance our understanding of the roles of specific COX enzymes in local O_2_ transport. However, to our knowledge, whether the COX‐2 isoform is obligatory for the regulation of the skeletal muscle microvascular oxygenation response to exercise has not been investigated.

Selective COX‐2 inhibitors are a class of non‐steroidal anti‐inflammatory drugs (NSAIDs) originally developed to spare the COX‐1 enzyme which protects the gastrointestinal lining, thereby minimizing side effects such as stomach ulcers and bleeding (McCormack, 2011; Wolfe et al., 1999). Celecoxib, the only U.S. Food and Drug Administration (FDA)‐approved COX‐2 inhibitor currently available in the USA, is commonly prescribed for patients needing pain and inflammation relief but who are at elevated risk for gastrointestinal side effects from traditional NSAIDs. This group includes individuals with osteoarthritis, rheumatoid arthritis, ankylosing spondylitis, dysmenorrhea, and those experiencing pain following surgery or injury (McCormack, 2011). Resolution of COX‐2's role in local oxygen delivery and utilization matching in health could thus provide valuable insights into how those processes may be disrupted in diseased states.

The purpose of the present study was to determine the effects of acute selective COX‐2 inhibition (SC2I) with celecoxib on skeletal muscle microvascular O_2_ delivery–utilization matching and exercise tolerance in healthy young individuals. Central and peripheral components of the O_2_ transport pathway were assessed during the transition from rest to moderate and severe intensity cycling exercise. Additionally, cuff occlusion (reactive hyperaemia) protocols evaluated the effects of acute SC2I on leg muscle microvascular oxygenation during periods of relative hypoxia. Considering the above, we hypothesized that acute SC2I would (1) reduce the plasma concentration of the stable PGI_2_ metabolite 6‐keto prostaglandin F_1α_ (6‐keto‐PGF_1α_); (2) impair leg muscle microvascular oxygenation during cuff occlusion protocols; (3) impair cardiorespiratory and skeletal muscle responses during the rest–exercise transition (i.e., arterial blood pressure, heart rate, pulmonary oxygen uptake (${\dot{V}}_{O_{2}}$) and leg muscle microvascular oxygenation); and thus (4) reduce exercise tolerance in healthy adults.

## 2 METHODS

Participants were recruited from Purdue University in West Lafayette, IN, USA. The population recruited was untrained men and women ranging from 18 to 35 years of age. Exclusion criteria included cardiovascular, respiratory and/or neuromuscular disease; orthopaedic limitation to cycling; hypertension (resting arterial blood pressure >140/90 mmHg); smoking; pregnancy; obesity (body mass index (BMI) > 30); type 1 or type 2 diabetes mellitus; hepatic or renal disease; hypersensitivity to celecoxib (selective COX‐2 inhibitor); and/or history of asthma or any allergic‐type reaction to aspirin or NSAIDs. Individuals who engaged in supervised physical activity or exercised more than three times per week during the 6 months preceding the screening were excluded from the study. Procedures were approved by Purdue University's Institutional Review Board (Project No. 1905022251) and conformed to the most recent version of the *Declaration of Helsinki*. All participants provided written informed consent before enrolment.

### 2.1 Experimental design

After 45 individuals were screened for eligibility, 18 met all inclusion criteria, and 12 participants completed the study. Among the six individuals who met the inclusion criteria but did not complete the study, three withdrew due to scheduling conflicts, two voluntarily chose to discontinue participation, and one ceased communication with the research team after completing at least one session. Following screening and enrolment, each participant reported to the laboratory on three different occasions within a 4‐ to 5‐week period. Participants were instructed to avoid strenuous exercise in the 24 h preceding each test session, refrain from eating for 3 h before exercise tests, and refrain from caffeine and alcohol intake for at least 12 and 24 h before each test, respectively. All experimental procedures were performed at approximately the same time of day (±2 h) for each participant.

During the first visit (visit 1), a ramp incremental test was performed for the determination of work rates to be employed during constant moderate and severe intensity tests in subsequent visits (i.e., visits 2 and 3) as described below. Participants were then assigned to a randomized, double‐blind, placebo‐controlled crossover design to either an oral dose of acute selective COX‐2 inhibitor (SC2I; 200 mg celecoxib, Pfizer Inc., New York) or a placebo (CON; cellulose). Celecoxib and placebo were processed and packaged to be visually and physically indistinguishable from one another. The celecoxib dose used in this study is typical to that prescribed for patients with osteoarthritis, rheumatoid arthritis and ankylosing spondylitis (Bannuru et al., 2019; McCormack, 2011; Simon et al., 1999; Stengaard‐Pedersen, 2004) and has been demonstrated to effectively and specifically inhibit the COX‐2 enzyme in healthy individuals (McAdam et al., 1999; Schwartz et al., 2008). During visits 2 and 3, CON and SC2I pills were taken approximately 2.5 h prior to the start of each experimental session. This time period is consistent with peak plasma concentrations of SC2I as reported previously (Pal et al., 2017; Paulson et al., 2000). A washout period of at least 4 days was allowed before crossing over to the other study condition.

### 2.2 Experimental procedures

All exercise tests were performed with the participant in the upright position on an electromagnetically braked cycle ergometer (Excalibur Sport, Lode, Netherlands) with a self‐selected pedal rate between 60 and 70 rpm. Participants were instructed to maintain their individual pedal rates throughout all exercise tests. Seat and handlebar height and configuration were recorded during visit 1 and replicated in subsequent visits. Strong verbal encouragement was provided during maximal incremental and severe constant work rate tests by the same investigator, who was blinded to the treatment condition. Exercise tests were terminated when participants signalled to stop pedalling or could not maintain the necessary pedal rate for ≥5 s despite strong verbal encouragement.

### 2.3 Visit 1: Incremental exercise test

Participants performed an incremental ramp test to determine the gas exchange threshold (GET), peak ${\dot{V}}_{O_{2}}$ and work rates to be employed in subsequent visits (i.e., visits 2 and 3). Baseline work rates (12–25 W) and ramp rates (12–25 W/min) were chosen individually to produce a test lasting approximately 8–12 min. Participants rested quietly on a chair for at least 5 min before transferring to the cycle ergometer. After resting data were collected for 2 min, participants exercised at a fixed load of 12–25 W for 1 min followed by the ramp incremental protocol. Heart rate was measured beat‐by‐beat using a sensor strapped to the thorax. Gas exchange data during the incremental test were averaged every 10 s. The GET was determined using the V‐slope and ventilatory equivalent methods (Beaver et al., 1986; Wasserman et al., 1973). Peak ${\dot{V}}_{O_{2}}$ was taken as the highest 10‐s average value attained before exercise termination. The work rates that would require 90% GET (moderate intensity) and 80% of peak work rate (severe intensity) were subsequently determined, taking into account the mean response time (MRT) for ${\dot{V}}_{O_{2}}$ during ramp exercise (Whipp et al., 1981).

### 2.4 Visits 2 and 3: Blood draws, cuff occlusion protocol and constant work rate exercise tests

As detailed above, participants ingested either SC2I or CON approximately 2.5 h before arrival at the laboratory. A venous blood sample was drawn for analysis of plasma concentrations of the stable PGI_2_ metabolite 6‐keto‐PGF_1α_ (McAdam et al., 1999; Phillips et al., 2004). Subsequently, a cuff occlusion (reactive hyperaemia) protocol was performed as described below. Participants then rested quietly on a chair for at least 5 min before transferring to the cycle ergometer. Following 2 min of resting data measurements, constant work rate exercise tests were initiated from rest to moderate and severe intensity work rates for the determination of arterial blood pressure and heart rate, pulmonary ${\dot{V}}_{O_{2}}$ and leg muscle oxygen saturation ($S_{tO_{2}}$) kinetics. The moderate intensity test was performed for a total of 6 min of exercise. After at least 30 min of passive recovery, the severe intensity test was performed until exhaustion.

### 2.5 Measurements

#### 2.5.1 Plasma concentrations of 6‐keto‐PGF_1α_

Upon arrival at the laboratory during visits 2 and 3, venous blood samples were collected in K_2_‐EDTA vacutainers (BD, Franklin Lakes, NJ, USA) and centrifuged at 1300 rpm for 15 min at 4°C. Plasma was extracted immediately and frozen at −80°C for subsequent analysis. Plasma concentration of 6‐keto‐PGF_1α_ was determined with a commercially available enzyme immunoassay kit (Cayman Chemical Co., Ann Arbor, MI, USA). Samples were diluted in a 1:2 ratio in assay diluent and were measured in duplicate. Absorbance was measured at 420 nm using a microplate reader (accuSkan, ThermoFisher Scientific, Waltham, MA, USA). 6‐keto‐PGF_1α_ assay sensitivity was 6 pg/mL as stated by the manufacturer.

#### 2.5.2 Central haemodynamics

Heart rate was monitored on a beat‐by‐beat basis from rest through moderate and severe exercise using a chest‐worn sensor strap (Polar H10, Polar Electro Oy, Kempele, Finland). Arterial blood pressure was determined via the auscultatory method.

#### 2.5.3 Pulmonary gas exchange and ventilation

Resting and exercising pulmonary gas exchange and ventilation were measured breath‐by‐breath using a calibrated, portable metabolic analyser (K4b2, Cosmed USA Inc., Concord, CA, USA). The following variables were measured: pulmonary ${\dot{V}}_{O_{2}}$, carbon dioxide output (${\dot{V}}_{CO_{2}}$), and ventilatory equivalents for O_2_ and CO_2_.

#### 2.5.4 Skeletal muscle microvascular oxygenation

Quadriceps muscle microvascular oxygenation of the dominant leg was measured using near‐infrared spectroscopy (NIRS; NIRO‐200NX, Hamamatsu Photonics, Hamamatsu City, Japan). Before probe placement, the area of the skin was prepared by shaving excess hair and cleansing with alcohol wipes to minimize impedance. The probes were secured to the skin over the vastus lateralis muscle with adhesive tape and an elastic bandage. The probe position was marked on the skin to ensure consistent placement on subsequent visits.

#### 2.5.5 Cuff occlusion protocol

A contoured cuff (CC22TM, D. E. Hokanson, Inc., Bellevue, WA, USA) was positioned proximally to the NIRS probe site around the upper thigh. Participants rested in a supine position while baseline NIRS data were collected for 2 min. The cuff was then rapidly inflated to 250 mmHg using an automatic rapid cuff inflator system (Hokanson E20 and AG101, D. E. Hokanson, Inc.) and maintained at this pressure for 5 min. Following this period, the cuff was quickly deflated, with participants remaining supine and at rest throughout the procedure. NIRS measurements during cuff occlusion and release (post‐occlusion) periods provided an index of skeletal muscle metabolic rate (O_2_ extraction) and microvascular reactivity, respectively (Rosenberry et al., 2018).

Baseline $S_{tO_{2}}$ was determined as the 30 s average before the onset of arterial cuff occlusion. The rate of oxygen desaturation during cuff occlusion (i.e., slope 1), referred to as the desaturation slope, provides an index of skeletal muscle metabolic rate (Doerschug et al., 2007). $S_{tO_{2}\min}$ is the lowest value achieved during ischaemia and was taken as a measure of ischaemic insult (stimulus for vasodilatation). The rate of oxygen resaturation after cuff release (i.e., slope 2), referred to as the reperfusion slope, indicates the recovery dynamics of microvascular oxygenation. $S_{tO_{2}\max}$ is the highest value obtained after cuff release. The area under the curve (AUC) was calculated during both the first and the second minute following cuff release (i.e., AUC_1min_ and AUC_2min_, respectively). The hyperaemic reserve was determined as the difference between $S_{tO_{2}\max}$ and baseline and reported as a percentage change.

### 2.6 Data analysis

Pulmonary ${\dot{V}}_{O_{2}}$, heart rate and $S_{tO_{2}}$ data points exceeding 4 standard deviations from the local mean were excluded from analysis (Lamarra et al., 1987). Responses were subsequently time‐aligned at exercise onset/cuff inflation, linearly interpolated every second (pulmonary ${\dot{V}}_{O_{2}}$), every 2 s (muscle $S_{tO_{2}}$) or averaged into 15‐s bins (heart rate) for each participant. Kinetic analyses were performed using non‐linear least‐squares regression (Marquardt–Levenberg; SigmaPlot 14.5; Systat Software, San Jose, CA, USA). The first 20 s of pulmonary ${\dot{V}}_{O_{2}}$ data following the onset of exercise (i.e., cardiodynamic phase) were removed from the analysis. Moderate and severe intensity ${\dot{V}}_{O_{2}}$ data were described using mono‐ and bi‐exponential models, respectively, as follows. One‐component:

$${\dot{V}}_{O_{2}\left(t\right)}={\dot{V}}_{O_{2}\mathrm{rest}}+A_{1}\left(1-e^{-\left(t-{\mathrm{TD}}_{1}/\tau_{1}\right)}\right)$$

Two‐component:

$${\dot{V}}_{O_{2}\left(t\right)}={\dot{V}}_{O_{2}\mathrm{rest}}+A_{1}\left(1-e^{-\left(t-{\mathrm{TD}}_{1}/\tau_{1}\right)}\right)+A_{2}\left(1-e^{-\left(t-{\mathrm{TD}}_{2}/\tau_{2}\right)}\right)$$

where ${\dot{V}}_{O_{2}(t)}$ indicates the ${\dot{V}}_{O_{2}}$ at a given time (*t*); ${\dot{V}}_{O_{2}\mathrm{rest}}$ is the resting value (30 s average) before exercise onset; *A*_1_ and *A*_2_ are the amplitudes for the exponential terms; τ_1_ and τ_2_ are the time constants; and TD_1_ and TD_2_ are the independent time delays. Similarly, individual muscle $S_{tO_{2}}$ profiles during moderate and severe exercise were described using mono‐ or bi‐exponential models as follows. One‐component:

$$S_{{\mathrm{tO}}_{2}\left(t\right)}=S_{{\mathrm{tO}}_{2}\mathrm{rest}}-A_{1}\left(1-e^{-\left(t-{\mathrm{TD}}_{1}/\tau_{1}\right)}\right)$$

Two‐component:

$$S_{{\mathrm{tO}}_{2}\left(t\right)}=S_{{\mathrm{tO}}_{2}\mathrm{rest}}-A_{1}\left(1-e^{-\left(t-{\mathrm{TD}}_{1}/\tau_{1}\right)}\right)+A_{2}\left(1-e^{-\left(t-{\mathrm{TD}}_{2}/\tau_{2}\right)}\right)$$

where subscripts *t* and rest, and parameters *A*, TD and τ are defined above. Goodness of fit and model selection were determined using three criteria: the coefficient of determination, the sum of squared residuals and visual inspection. The overall dynamics of the ${\dot{V}}_{O_{2}}$ and $S_{tO_{2}}$ profiles, represented by the MRT, were determined as follows (Macdonald et al., 1997). One‐component:

$$\mathrm{MRT}\;=TD_{1}+\tau_{1}$$

Two‐component:

$$\mathrm{MRT}=\left(A_{1}/A_{\mathrm{total}}\right)\;\left(TD_{1}+\tau_{1}\right)+\left(A_{2}/A_{\mathrm{total}}\right)\left(TD_{2}+\tau_{2}\right)$$

where *A*_1_ and *A*_2_, TD_1_ and TD_2_, and τ_1_ and τ_2_ are defined above, and *A*_total_ is the total change in ${\dot{V}}_{O_{2}}$ (*A*_1_ + *A*_2_) when using the two‐component model. The MRT analysis for $S_{tO_{2}}$ during moderate and severe exercise was constrained to the one‐component model, given that the inclusion of the emergent second component underestimates the speed of fall during the onset of muscle contractions (Hirai et al., 2009).

### 2.7 Statistical analysis

All data analyses were performed using commercially available software (SigmaPlot 14.5; Systat Software), with the assessor blinded to the experimental condition. Comparisons of plasma concentrations of 6‐keto‐PGF_1α_, peak ${\dot{V}}_{O_{2}}$, NIRS data during cuff occlusion protocols, and endurance time were performed using a two‐tailed paired Student's *t*‐test. Arterial blood pressure (systolic, diastolic and mean arterial pressure (MAP)), heart rate, pulmonary ${\dot{V}}_{O_{2}}$ kinetics and leg muscle $S_{tO_{2}}$ kinetics were analysed using two‐way repeated‐measures ANOVA. *Post hoc* analyses were conducted using the Student−Newman−Keuls test. Due to methodological shortcomings, the available sample sizes for the following measurements were limited as follows: 6‐keto‐PGF_1α_ plasma concentration (*n *= 8); and moderate intensity ${\dot{V}}_{O_{2}}$, $S_{tO_{2}}$, and cuff occlusion NIRS data (*n *= 11). Data are presented as means (SD). Significance was accepted at *P *< 0.05.

## 3 RESULTS

**FIGURE 1:**
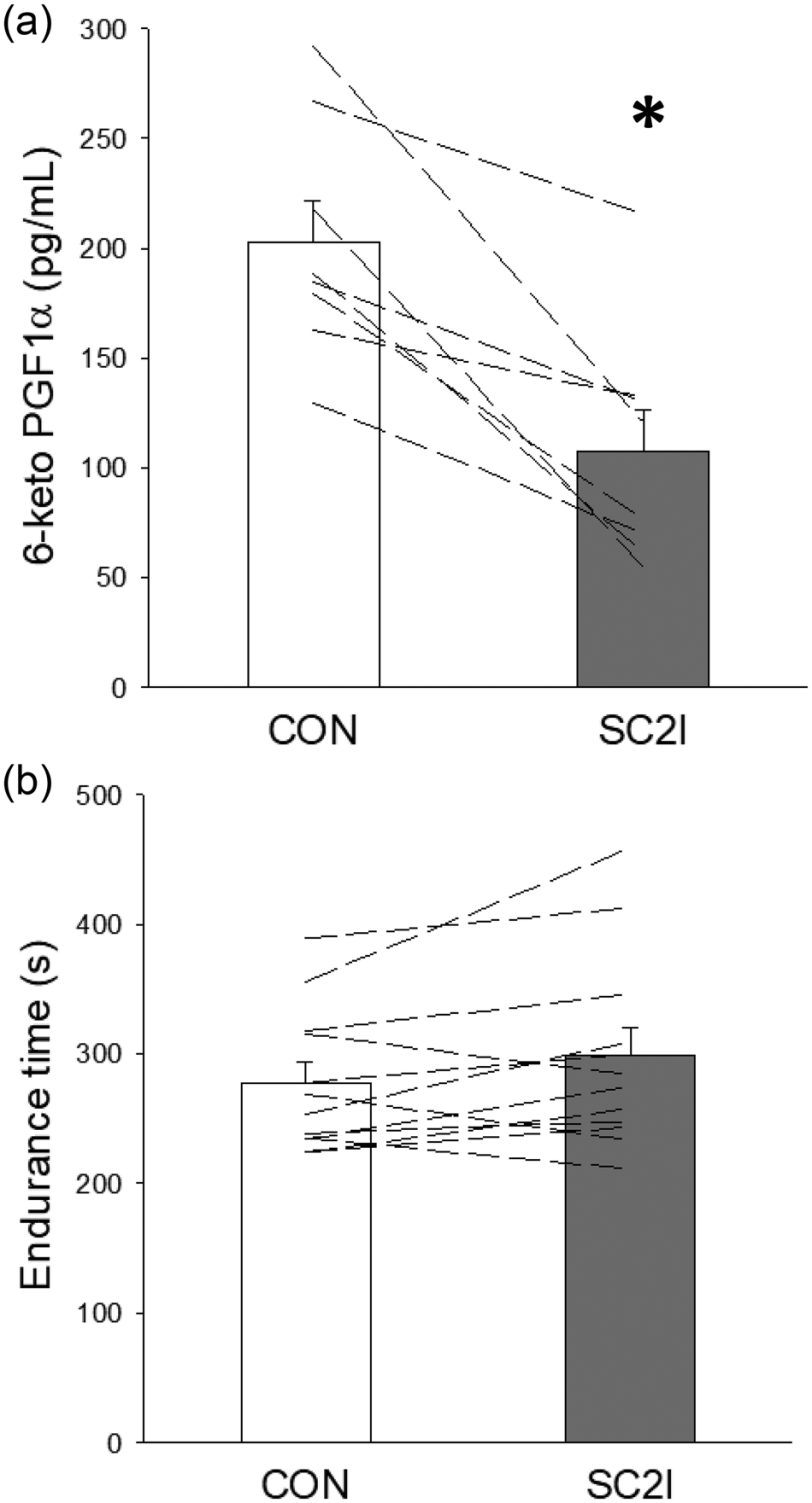
(a) Group mean and individual plasma 6‐keto‐PGF_1α_ concentrations under CON (*n* = 8) and SC2I (*n* = 8). (b) Group mean and individual endurance times during severe intensity exercise under CON (*n* = 12) and SC2I (*n* = 12). Values are means (SD). *P = 0.002 versus CON. CON, control; SC2I, selective COX‐2 inhibition.

Participant characteristics and responses to incremental exercise are shown in Table 1. Work rates utilized during moderate and severe exercise tests were 65 (21) and 144 (36) W, respectively. Resting heart rate during the incremental test was higher than typically observed in untrained healthy adults (96 ± 14 bpm), which may be attributed to participant anxiety and the associated anticipatory response. Nonetheless, individual resting values were consistent across all three visits, and there were no significant differences in heart rate between conditions (*P *> 0.05). As illustrated in Figure 1a, SC2I significantly reduced the plasma concentrations of the stable PGI_2_ metabolite 6‐keto‐PGF_1α_ in all cases compared with CON (*P* = 0.002). There were no significant differences in exercise tolerance between CON and SC2I (Figure 1b; *P *> 0.05).

**TABLE 1:** Participant characteristics and responses to ramp‐incremental exercise.

Characteristic	Value
Demographic/anthropometric	
Male:female (*n*)	4:8
Age (years)	25 (4)
Weight (kg)	67.9 (12.3)
Height (cm)	169 (6)
BMI (kg/m^2^)	23.6 (3.0)
Incremental exercise	
Ramp rate (W/min)	17.9 (4.4)
Peak work rate (W)	177 (41)
Peak ${\dot{V}}_{O_{2}}$	
L/min	1.85 (0.37)
mL/kg/min	27.70 (5.72)
${\dot{V}}_{O_{2}}$ at the GET	
L/min	1.10 (0.23)
mL/kg/min	17.07 (4.25)
Heart rate, bpm	
Rest	96 (14)
Peak exercise	192 (5)

*Note*: When noted, values are means (SD). *n* = 12. Abbreviations: BMI, body mass index; GET, gas exchange threshold; ${\dot{V}}_{O_{2}}$, oxygen uptake.

**FIGURE 2:**
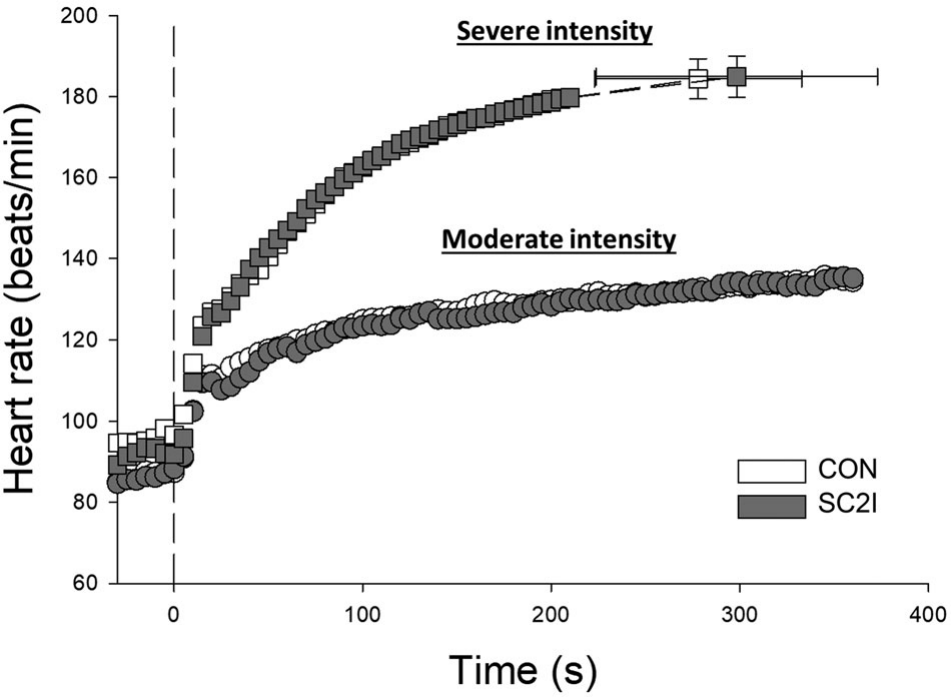
Group mean heart rate from rest to moderate (CON, *n* = 12; SC2I, *n* = 12) and severe (CON, *n* = 12; SC2I, *n* = 12) intensity exercise under CON and SC2I. Values are means (SD). Error bars omitted for clarity (except for maximal values during severe intensity exercise). Exercise started at time 0. CON, control; SC2I, selective COX‐2 inhibition.

As illustrated in Figure 2, there were no significant differences in heart rate during the transition from rest to moderate or severe exercise between conditions (*P *> 0.05). Similarly, there were no differences in blood pressure (systolic, diastolic or MAP) at rest or during moderate or severe exercise between conditions (Table 2, *P *> 0.05 for all).

**TABLE 2:** Systolic, diastolic and mean arterial pressure at rest and during moderate and severe intensity exercise under CON and SC2I.

	Moderate intensity		Severe intensity	
	CON	SC2I	CON	SC2I
Systolic blood pressure (mmHg)				
Rest	116 (8)	115 (8)	117 (8)	117 (8)
Exercise	130 (7)	133 (12)	151 (14)	149 (17)
Diastolic blood pressure (mmHg)				
Rest	78 (6)	78 (7)	79 (6)	79 (7)
Exercise	77 (5)	78 (6)	77 (5)	77 (7)
MAP (mmHg)				
Rest	91 (6)	90 (7)	92 (7)	92 (7)
Exercise	95 (5)	96 (6)	102 (8)	101 (7)

*Note*: Values are means (SD). Moderate intensity: CON, *n* = 12; SC2I, *n* = 12. Severe intensity: CON, *n* = 12; SC2I, *n* = 12. Abbreviations: CON, control; MAP, mean arterial pressure; SCI21, selective COX‐2 inhibition.

**FIGURE 3:**
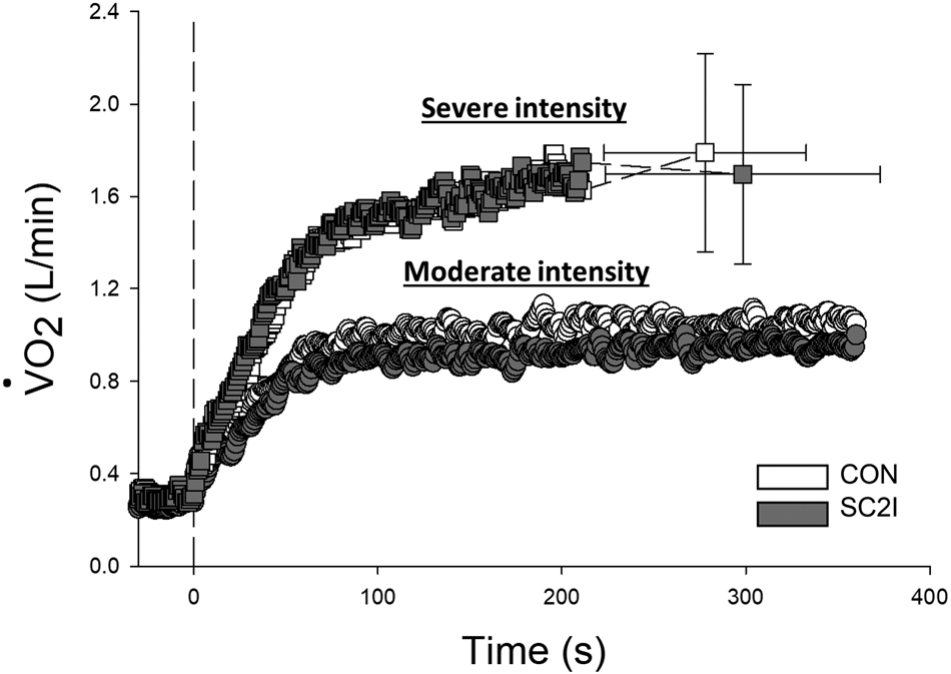
Group mean pulmonary ${\dot{V}}_{O_{2}}$ from rest to moderate (CON, *n* = 11; SC2I, *n* = 11) and severe (CON, *n* = 12; SC2I, *n* = 12) intensity exercise under CON and SC2I. Values are means (SD). Error bars omitted for clarity (except for maximal values during severe intensity exercise). Exercise started at time 0. CON, control; SC2I, selective COX‐2 inhibition; ${\dot{V}}_{O_{2}}$, oxygen uptake.

Pulmonary ${\dot{V}}_{O_{2}}$ responses from rest to moderate and severe intensity exercise following CON and SC2I administration are shown in Figure 3, and the parameters derived from model fits are presented in Table 3. No significant differences in ${\dot{V}}_{O_{2}}$ kinetics were detected between conditions (*P *> 0.05 for all parameters). Similarly, there were no differences in peak ${\dot{V}}_{O_{2}}$ during severe exercise between conditions (Figure 3, *P *> 0.05).

**TABLE 3:** Pulmonary ${\dot{V}}_{O_{2}}$ kinetics from rest to moderate and severe intensity exercise under CON and SC2I.

	Moderate intensity		Severe intensity	
	CON	SC2I	CON	SC2I
${\dot{V}}_{O_{2}\mathrm{rest}}$ (L/min)	0.29 (0.09)	0.27 (0.19)	0.37 (0.11)	0.33 (0.07)
τ_1_ (s)	31.3 (9.1)	29.2 (13.1)	32.8 (10.8)	30.9 (13.6)
*A*_1_ (L/min)	0.76 (0.22)	0.67 (0.25)	1.33 (0.32)	1.35 (0.34)
TD_1_ (s)	6.3 (7.9)	9.2 (7.5)	9.3 (5.4)	8.9 (6.2)
τ_2_ (s)	—	—	53.0 (33.4)	63.4 (31.0)
*A*_2_ (L/min)	—	—	0.27 (0.19)	0.23 (0.09)
TD_2_ (s)	—	—	148.1 (16.2)	141.7 (31.5)
MRT (s)	37.6 (7.2)	38.3 (10.3)	69.3 (13.4)	65.3 (16.5)

*Note*: Values are means (SD). Moderate intensity exercise: CON, *n* = 11; SC2I, *n* = 11. Severe intensity exercise: CON, *n* = 12; SC2I, *n* = 12. All moderate intensity profiles were analysed with the one‐component model, while all severe intensity profiles were analysed with the two‐component model. Abbreviations: *A*_1_, phase II amplitude; *A*_2_, slow component amplitude; CON, control; MRT, mean response time; TD_1_, phase II time delay; TD_2_, slow component time delay; ${\dot{V}}_{O_{2}}$, oxygen uptake; ${\dot{V}}_{O_{2}\mathrm{rest}}$, resting ${\dot{V}}_{O_{2}}$; τ_1_, phase II time constant; τ_2_, slow component time constant; SCI21, selective COX‐2 inhibition.

**FIGURE 4:**
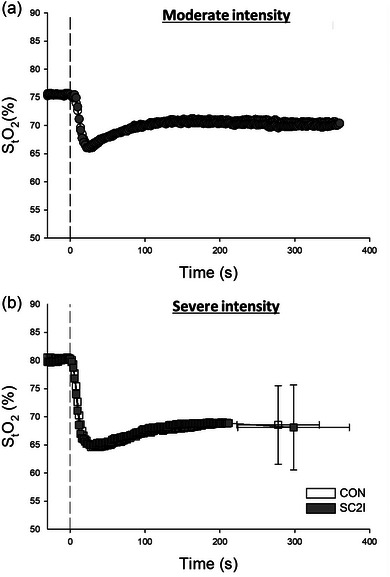
Group mean $S_{tO_{2}}$ from rest to moderate (a; CON, *n* = 11; SC2I, *n* = 11) and severe intensity (b; CON, *n* = 12; CON, *n* = 12) exercise under CON and SC2I. Values are means (SD). Error bars omitted for clarity (except for maximal values during severe intensity exercise). Exercise started at time 0. CON, control; SC2I, selective COX‐2 inhibition; $S_{tO_{2}}$, skeletal muscle oxygenation.

Leg muscle $S_{tO_{2}}$ responses from rest to moderate and severe intensity exercise under CON and SC2I are shown in Figure 4, and the parameters derived from model fits are presented in Table 4. There were no significant differences in muscle $S_{tO_{2}}$ kinetics between conditions (*P *> 0.05 for all parameters).

**TABLE 4:** $S_{tO_{2}}$ from rest to moderate and severe intensity exercise under CON and SC2I.

	Moderate intensity		Severe intensity	
	CON	SC2I	CON	SC2I
$S_{tO_{2}\mathrm{rest}}$ (%)	75.7 (6.7)	76.0 (4.6)	80.4 (5.9)	79.9 (4.6)
τ_1_ (s)	9.8 (7.3)	8.0 (5.4)	5.4 (1.4)	4.9 (1.9)
*A*_1_ (%)	13.1 (5.3)	11.9 (6.4)	16.1 (8.0)	14.9 (7.5)
TD_1_ (s)	7.4 (5.2)	6.9 (4.9)	5.7 (2.7)	5.2 (2.1)
τ_2_ (s)	47.1 (31.8)	41.5 (28.1)	35.1 (17.2)	38.3 (31.9)
*A*_2_ (%)	7.1 (4.4)	11.9 (15.8)	6.3 (5.0)	6.3 (5.2)
TD_2_ (s)	24.9 (8.0)	30.6 (18.8)	60.5 (39.4)	49.0 (16.1)
MRT (s)	17.2 (8.3)	14.9 (3.2)	11.1 (3.4)	10.2 (2.2)

*Note*: Values are means (SD). Moderate intensity exercise: CON, *n* = 11; SC2I, *n* = 11. Severe intensity exercise: CON, *n* = 12; SC2I, *n* = 12. Most moderate and severe intensity profiles were analysed with the two‐component model (moderate intensity: CON = 11/11, SC2I = 10/11; severe intensity: CON = 10/12; SC2I = 8/12). Abbreviations: A_1_, amplitude of the first component; A_2_, amplitude of the second component; CON, control; MRT, mean response time; $S_{tO_{2}}$, skeletal muscle microvascular oxygenation; $S_{tO_{2}\mathrm{rest}}$, resting $S_{tO_{2}}$; TD_1_, time delay of the first component; TD_2_, time delay of second component; τ_1_, time constant for the first component; τ_2_, time constant for the second component.

**FIGURE 5:**
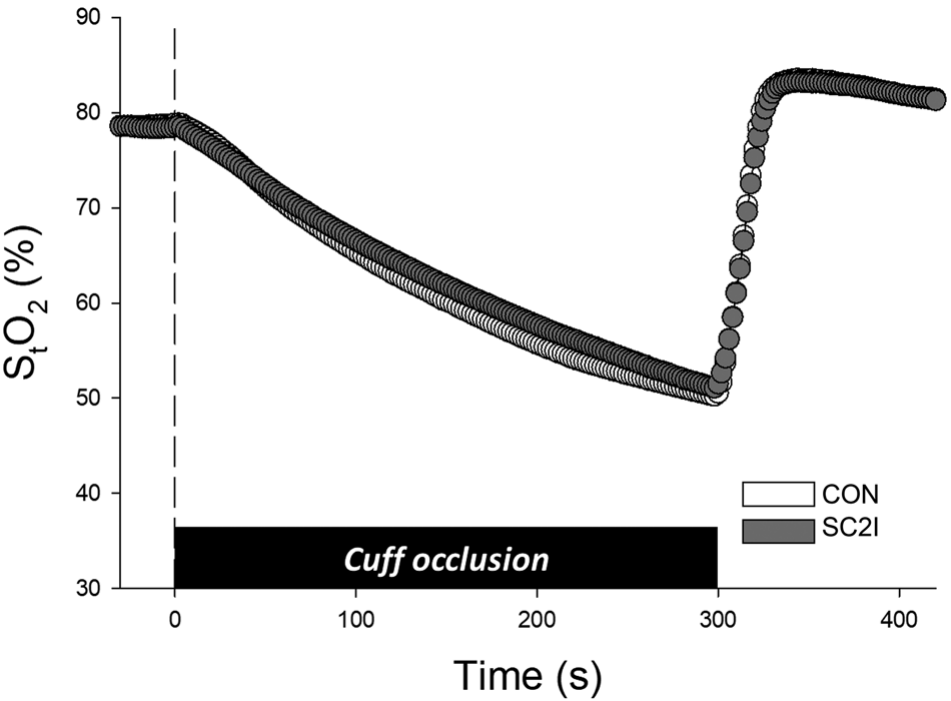
Group mean $S_{tO_{2}}$ during cuff occlusion (reactive hyperaemia) protocols under control (CON, *n* = 11) and selective COX‐2 inhibition (SC2I, *n* = 11). Error bars omitted for clarity. Cuff inflation and deflation initiated at time 0 and 300 s, respectively. CON, control; SC2I, selective COX‐2 inhibition; $S_{tO_{2}}$, skeletal muscle oxygenation.

Leg muscle $S_{tO_{2}}$ responses during cuff occlusion protocols are shown in Figure 5 and Table 5. There were no significant differences in muscle $S_{tO_{2}}$ before, during or after occlusion between CON and SC2I (*P *> 0.05 for all parameters).

**TABLE 5:** $S_{tO_{2}}$ during cuff occlusion (reactive hyperaemia) protocols.

	CON	SC2I
Baseline $S_{tO_{2}}$ (%)	78.9 (2.4)	77.8 (3.7)
Slope 1 (%/s)	−0.08 (0.03)	−0.08 (0.03)
$S_{tO_{2}\min}$ (%)	55.1 (8.4)	54.3 (9.8)
Slope 2 (%/s)	1.17 (0.44)	1.15 (0.32)
$S_{tO_{2}\max}$ (%)	83.2 (2.6)	82.9 (2.6)
Hyperaemic reserve (%)	4.4 (3.2)	5.0 (3.5)
1 min AUC (% s)	4590 (144)	4542 (258)
2 min AUC (% s)	9504 (293)	9442 (421)

*Note*: Values are means (SD). CON, *n* = 11; SC2I, *n* = 11. Abbreviations: AUC, area under the $S_{tO_{2}}$ curve post‐cuff release; baseline $S_{tO_{2}}$, pre‐occlusion $S_{tO_{2}}$; $S_{tO_{2}\max}$, highest tissue oxygen saturation achieved upon cuff release; hyperaemic reserve, difference between $S_{tO_{2}\max}$ and baseline $S_{tO_{2}}$; $S_{tO_{2}\max}$, lowest $S_{tO_{2}}$ achieved during cuff occlusion.

## 4 DISCUSSION

This is the first evaluation of the effects of acute SC2I on skeletal muscle microvascular O_2_ delivery–utilization matching and exercise tolerance in healthy adults. The significant reduction in the plasma concentration of the stable PGI_2_ metabolite 6‐keto‐PGF_1α_ with SC2I did not translate to changes in resting or exercising heart rate, arterial blood pressure, pulmonary ${\dot{V}}_{O_{2}}$ or skeletal muscle microvascular $S_{tO_{2}}$. In addition, SC2I did not affect leg muscle microvascular responses during cuff occlusion protocols. Taken together, these results do not support the hypothesis that acute SC2I would impair resting or contracting skeletal muscle microvascular O_2_ delivery–utilization matching and, consequently, reduce exercise tolerance.

### 4.1 SC2I and plasma 6‐keto‐PGF_1α_

Plasma concentrations of 6‐keto‐PGF_1α_ were diminished by ∼47% with SC2I compared to CON (Figure 1a), indicating that acute administration of celecoxib effectively reduced PGI_2_ production. These findings align with previous investigations that observed reductions in 6‐keto‐PGF_1α_ after SC2I in humans (Graff et al., 2007; McAdam et al., 1999; Stichtenoth et al., 2005) and animal models (Hsieh et al., 2008; Tunctan et al., 2010; Wong et al., 2001). As noted above, the celecoxib dosage used herein (1) has been demonstrated to effectively and specifically inhibit the COX‐2 enzyme in healthy individuals (McAdam et al., 1999; McCormack, 2011; Schwartz et al., 2008), and (2) constitutes the recommended therapeutic dosage, which has been shown to confer clinically meaningful effects in patients with osteoarthritis, rheumatoid arthritis and ankylosing spondylitis (McCormack, 2011; Simon et al., 1999). Nonetheless, while this 6‐keto‐PGF_1α_ reduction is consistent with COX‐2 inhibition, contributions from COX‐1 to residual PGI_2_ production cannot be excluded under the present conditions.

The release of 6‐keto‐PGF_1α_ is associated with endothelium‐mediated vasodilatation during hypoxia and skeletal muscle contractions (Busse et al., 1984; Win & Marshall, 2005). Notably, exercise‐induced increases in 6‐keto‐PGF_1α_ concentration have been shown to correlate with maximal ${\dot{V}}_{O_{2}}$ in healthy young individuals (Zoladz et al., 2009), underscoring the potential role of PGI_2_ in muscle oxygen transport and exercise tolerance. Non‐selective COX inhibition in humans is capable of reducing 6‐keto‐PGF_1α_ and altering cardiovascular responses, resulting in elevated mean arterial pressure and limb vascular resistance during resistive breathing with ibuprofen (Smith et al., 2017), and lowered skeletal muscle blood flow with indomethacin or aspirin (Duffy et al., 1998; Wilson & Kapoor, 1993). In contrast, the reduction in 6‐keto‐PGF_1α_ following SC2I with celecoxib herein (Figure 1a) was not accompanied by changes in resting or exercising blood pressure or heart rate (Table 2 and Figure 2, respectively). This disparity in cardiovascular responses to reductions in 6‐keto‐PGF_1α_ may result from the use of non‐selective versus selective COX inhibition and their distinct impact on the TxA_2_/PGI_2_ balance as mentioned above. SC2I, as employed in the present study, enables a more targeted exploration of vasodilatory prostanoids like PGI_2_ thereby advancing our understanding of their specific role in skeletal muscle oxygen transport and exercise performance.

### 4.2 SC2I: Moderate and severe intensity exercise tests

#### 4.2.1 Central haemodynamics

COX products are known to stimulate sympathetic nerve activity (Ragan et al., 2007; Rotto & Kaufman, 1988), but the effects of non‐selective inhibition on central haemodynamic responses during exercise remain largely inconsistent. Some studies have reported lower mean arterial pressure (MAP) during exercise following non‐selective COX inhibition (Cowley et al., 1985; Cui et al., 2008; Stebbins et al., 1986), while others have found no significant changes (Duffy et al., 1999; Middlekauff & Chiu, 2004; Momen et al., 2008; Naylor et al., 1999; Wilson & Kapoor, 1993; Win & Marshall, 2005). Similarly, non‐selective COX inhibition has generally produced mixed effects on heart rate during exercise, with at least one study reporting a significant decrease with indomethacin but not aspirin (Cowley et al., 1985), while several others observed no significant differences (Middlekauff & Chiu, 2004; Momen et al., 2008; Shoemaker et al., 1996; Stebbins et al., 1986; Wilson & Kapoor, 1993). To the best of our knowledge, this is the first study to examine the effects of acute SC2I on central haemodynamics during the transition from rest to moderate and severe intensity exercise. As shown in Table 2 and Figure 2, acute SC2I with celecoxib had no effects on arterial blood pressure or heart rate during moderate or severe exercise in healthy young individuals.

#### 4.2.2 Skeletal muscle oxygen utilization

Earlier research using non‐selective inhibitors has reported varied results on the role of COX products in regulating skeletal muscle O_2_ utilization. Some studies support their involvement (Boushel et al., 2012), while others do not (Mortensen et al., 2007; Young & Sparks, 1980). This divergence could relate, at least in part, to different techniques used to assess O_2_ utilization, muscle contraction protocols (e.g., distinct muscle groups and exercise intensities), non‐selective COX inhibitor classes and doses, and the extent of COX inhibition. For instance, Boushel et al. investigated the independent and combined effects of nitric oxide synthase and non‐selective COX blockade (with L‐NAME and indomethacin, respectively) on mitochondrial respiration following knee extension exercise. They reported a decrease in state 3 mitochondrial respiration primarily at complex I of the respiratory chain after arterial infusion of indomethacin alone, thereby suggesting a role for COX products in the regulation of mitochondrial O_2_ consumption (Boushel et al., 2012). Moreover, exercise‐induced PGI_2_ release (assessed as plasma 6‐keto‐PGF_1α_ concentration) appears to be correlated with maximal ${\dot{V}}_{O_{2}}$ in healthy individuals (Zoladz et al., 2009) as noted above. In the present study, pulmonary ${\dot{V}}_{O_{2}}$ responses at rest or during moderate or severe intensity exercise were not different between SC2I and CON (Figure 3 and Table 3). Similarly, peak ${\dot{V}}_{O_{2}}$ was not different between experimental conditions (Figure 3). Our findings align with studies reporting no change in pulmonary ${\dot{V}}_{O_{2}}$ during acute non‐selective COX inhibition with indomethacin in both humans and animal models (Mortensen et al., 2007; Young & Sparks, 1980), thus supporting that COX‐2 is not obligatory for the regulation of O_2_ utilization during exercise.

#### 4.2.3 Skeletal muscle oxygen delivery

Previous studies have evaluated the role of prostanoids in regulating skeletal muscle blood flow via non‐selective COX inhibition but, to our knowledge, whether COX‐2 per se modulates skeletal muscle microvascular O_2_ delivery‐utilization matching had not been determined. Notably, non‐selective COX inhibition has yielded conflicting findings regarding muscle blood flow responses at rest and during contractions. Heraczynska‐Cedro and colleagues (Herbaczynska‐Cedro et al., 1976; Janczewska & Herbaczyńska‐Cedro, 1974) were the first to provide direct evidence, using a canine model, that muscle contractions increase prostaglandin‐like substances. Moreover, they reported that the non‐selective COX inhibitor indomethacin abolished prostaglandin release and decreased the vasodilatory response to exercise. Duffy et al. administered the non‐selective COX inhibitor aspirin to determine the influence of vasodilator prostanoids on resting and exercising forearm blood flow in humans and reported a decrease in resting and peak exercise blood flow (Duffy et al., 1999, 1998). Schrage et al. employed the non‐selective inhibitor ketorolac and noted that vasodilating prostanoids, as well as nitric oxide, contribute independently to exercise hyperaemia (Schrage et al., 2004). Murrant et al. used indomethacin with the hamster cremaster preparation and found that prostanoids participate in arteriolar vasodilatation to muscle contractions independently of adenosine release (Murrant et al., 2014). Pertinent to the present study, the abovementioned data from Murrant et al. highlight the potential role of vasodilating prostanoids within the skeletal muscle microvascular network. Taken together, these and other studies using non‐selective inhibitors (Cowley et al., 1985; Kilbom & Wennmalm, 1976; Wilson & Kapoor, 1993; Win & Marshall, 2005) support that COX metabolites regulate blood flow (and thus O_2_ delivery) during muscle contractions.

Nevertheless, it is important to note that other studies employing non‐selective COX inhibition suggest that vasodilatory prostanoids do not play an obligatory role in the regulation of exercise hyperaemia. As mentioned above, such discrepancy could result, at least in part, from experimental differences including techniques used to assess vasodilatation or muscle blood flow, exercise protocols (e.g., distinct muscle groups and exercise intensities), non‐selective COX inhibitor classes and doses, and the extent of COX inhibition. Young and Sparks reported no change in contracting muscle blood flow following indomethacin administration in the dog despite significant reductions in vascular conductance at rest (Young & Sparks, 1980). Shoemaker et al. evaluated the effects of the non‐selective COX inhibitor ibuprofen at rest and during dynamic handgrip exercise and observed no changes in forearm artery diameter, mean blood velocity or blood flow in healthy adults (Shoemaker et al., 1996). Furthermore, Lopez et al. investigated forearm blood flow in healthy adults at rest and during ischaemic handgrip exercise and found no significant effects of arterial ketorolac infusion compared to saline (Lopez et al., 2013). The present findings align with the aforementioned studies and others (Beaty & Donald, 1979; Casey & Joyner, 2011; Crecelius et al., 2011; Einstein & Goodman, 1980; Mortensen et al., 2007; Nowak & Wennmalm, 1979), which generally show no impact of non‐selective COX inhibitors on the regulation of skeletal muscle blood flow at rest or during contractions. Notwithstanding the significant decrease in 6‐keto‐PGF_1α_ following acute SC2I with celecoxib herein (Figure 1a), no differences were detected in vastus lateralis microvascular $S_{tO_{2}}$ at rest or during moderate or severe exercise between CON and SC2I (Figure 4 and Table 4). The latter findings help explain the lack of changes in endurance time with SC2I (Figure 1b), given that skeletal muscle microvascular oxygenation is a key determinant of exercise tolerance (Poole & Jones, 2012).

The vascular system is well‐known for its considerable redundancy, with multiple vasodilatory agents contributing to O_2_ delivery during muscular contractions and compensatory mechanisms activated when one or more pathways are attenuated or blocked (Joyner & Casey, 2015; Joyner & Wilkins, 2007). Such redundancy may account for the lack of significant differences in the physiological outcomes assessed herein despite the lowered 6‐keto‐PGF_1α_ with SC2I in healthy individuals. It is possible that other vasodilatory agents (e.g., nitric oxide, adenosine, endothelium‐derived hyperpolarization factors, among others) may aid in the matching of skeletal muscle O_2_ delivery and utilization at least under the conditions evaluated herein. The present results thus indicate that COX‐2 products are not obligatory for regulating skeletal muscle microvascular oxygenation at rest or during moderate or severe intensity exercise in healthy adults. Whether acute SC2I may impair skeletal muscle O_2_ delivery‐utilization matching and, consequently, exercise tolerance in individuals presenting with vascular dysfunction and compromised redundant control mechanisms remains to be determined (Poole et al., 2021, 2012).

### 4.3 SC2I: Cuff occlusion protocols

NIRS‐derived measurements during and after circulatory occlusion were used herein to assess skeletal muscle metabolic rate (O_2_ extraction) and microvascular reactivity, respectively (Rosenberry et al., 2018). As noted earlier, the vasodilatory role mediated by prostaglandins such as PGI_2_ may become more pronounced under conditions of reduced O_2_ availability (Busse et al., 1984; Michiels et al., 1993). However, contrary to our hypothesis, acute SC2I with celecoxib had no impact on leg muscle microvascular oxygenation during occlusion protocols (Figure 5 and Table 5). These findings contrast with the potential modulatory effects of non‐selective COX inhibition on peripheral vascular control reported previously (Engelke et al., 1996; Nowak & Wennmalm, 1979). For instance, Nowak and Wennmalm found that indomethacin lowered both the peak and duration of the reactive hyperaemic response in the human forearm and calf muscles (Nowak & Wennmalm, 1979). Engelke et al. corroborated those findings by reporting a significant reduction in peak forearm blood flow upon cuff deflation with ibuprofen administration compared to control (Engelke et al., 1996). While the abovementioned studies suggest that the effects of non‐selective COX inhibition during occlusion protocols primarily reflect the blockade of vasodilator prostaglandins, other evidence indicates that inhibition of vasoconstrictor prostaglandins plays a more important role in shaping vasoregulatory responses. Accordingly, Faisal et al. reported that ibuprofen elevated forearm blood flow following 15 min of vascular occlusion in healthy young individuals (Faisal et al., 2010). A study by Naylor et al. indicated that non‐selective COX inhibition with indomethacin or ibuprofen had negligible effects following circulatory occlusion alone, but augmented reactive hyperaemia after ischaemic exercise (Naylor et al., 1999). Further adding to the complexity of COX's role in microvascular function, Casey and Joyner showed that the non‐specific COX inhibitor ketorolac did not impact the compensatory vasodilatation in the contracting human forearm during hypoperfusion evoked by intra‐arterial balloon inflation (Casey & Joyner, 2011). Collectively, these studies underscore the value of selective COX inhibition in isolating the roles of specific enzymes and their products in matching skeletal muscle oxygen delivery‐utilization. Results from our occlusion experiments thus suggest that COX‐2 is not obligatory for the regulation of skeletal muscle microvascular oxygenation in healthy adults (Figure 5 and Table 5).

### 4.4 Experimental considerations

Notwithstanding the robust experimental design of the current study (i.e., a randomized, double‐blind, placebo‐controlled, crossover trial), the modest sample size might limit the generalizability of the results. Future studies should also examine how sex and fitness level may modulate the physiological effects of SC2I. Given that selectivity for COX‐2 exists on a continuum, the possibility of some degree of COX‐1 inhibition alongside COX‐2 inhibition cannot be ruled out under the present conditions. Moreover, assessment of 6‐keto‐PGF_1α_ was conducted at rest, limiting the ability to evaluate the effects of celecoxib on its dynamic response during or after exercise. While the current results indicate that celecoxib had no impact on multiple outcomes despite a reduction in plasma 6‐keto‐PGF_1α_, direct assessments of skeletal muscle blood flow are warranted to fully characterize the haemodynamic effects of SC2I.

NIRS signals can be influenced by the differential pathlength factor, which accounts for the scattering of light as it travels through tissue and may vary with adipose tissue thickness (ATT, Barstow, 2019; Pirovano et al., 2021). Although ATT was not measured in this study, it has been proposed that correction for ATT may not be necessary when using $S_{tO_{2}}$ as the primary NIRS outcome, as was the case here (Barstow, 2019). Moreover, all participants in the current study were non‐obese (BMI range: 19.2–29.3 kg/m^2^), further reducing the potential for ATT to have considerably affected the NIRS signal or introduced bias between conditions (i.e., CON or SC2I).

### 4.5 Summary and conclusions

This study examined for the first time the effects of acute SC2I via oral celecoxib on cardiorespiratory and skeletal muscle microvascular function at rest and during moderate and severe intensity exercise in healthy young individuals. Although reducing plasma concentrations of the stable PGI_2_ metabolite 6‐keto‐PGF_1α_, SC2I had no effect on resting or exercising arterial blood pressure, heart rate, pulmonary ${\dot{V}}_{O_{2}}$, leg muscle microvascular oxygenation or exercise performance (time to exhaustion or peak ${\dot{V}}_{O_{2}}$). These results indicate that COX‐2 is not obligatory for the control of central or peripheral determinants of O_2_ transport during the rest–exercise transition and, therefore, does not modulate exercise tolerance in healthy adults.

## AUTHOR CONTRIBUTIONS

Michael D. Belbis, Michael J. Holmes, Carly Day, Nicole L. Noel, Bruno T. Roseguini, and Daniel M. Hirai conceived and designed research. Michael D. Belbis, Michael J. Holmes, Joseph Yao, Christopher W. Kinnick, and Christopher K. Kargl performed experiments. Michael D. Belbis, Michael J. Holmes, Joseph Yao, Christopher W. Kinnick, Christopher K. Kargl, and Daniel M. Hirai analysed data. Michael D. Belbis, Michael J. Holmes, Christopher K. Kargl, Timothy P. Gavin, Bruno T. Roseguini, and Daniel M. Hirai interpreted results of experiments. Michael D. Belbis, Joseph Yao, Christopher W. Kinnick, and Daniel M. Hirai prepared figures. Michael D. Belbis and Daniel M. Hirai drafted the manuscript. Michael D. Belbis, Michael J. Holmes, Joseph Yao, Christopher W. Kinnick, Christopher K. Kargl, Timothy P. Gavin, Bruno T. Roseguini, and Daniel M. Hirai edited and revised the manuscript. All authors have read and approved the final version of this manuscript and agree to be accountable for all aspects of the work in ensuring that questions related to the accuracy or integrity of any part of the work are appropriately investigated and resolved. All persons designated as authors qualify for authorship, and all those who qualify for authorship are listed.

## CONFLICT OF INTEREST

None decalred.

## FUNDING INFORMATION

None.

## Data Availability

The data that support the findings of this study are available from the corresponding author upon reasonable request.
